# A high-throughput drug combination screen identifies an anti-glioma synergism between TH588 and PI3K inhibitors

**DOI:** 10.1186/s12935-020-01427-0

**Published:** 2020-07-23

**Authors:** Zhen Chen, Chao Chen, Tingting Zhou, Chao Duan, Qianqian Wang, Xiaohui Zhou, Xia Zhang, Fangrong Wu, Yunfen Hua, Fan Lin

**Affiliations:** 1 Department of Cell Biology, School of Basic Medical Sciences, Nanjing Medical University, XueHai Building A111, 101 Longmian Avenue, Nanjing, Jiangning District China; 2 Department of Oncology, The First Affiliated Hospital of Nanjing Medical University, Nanjing, China; 3 College of Pharmaceutical Science, Zhejiang University of Technology, Hangzhou, China; 4 Institute for Brain Tumors, Key Laboratory of Rare Metabolic Diseases, The Affiliated Cancer Hospital of Nanjing Medical University; Key Laboratory of Human Functional Genomics of Jiangsu Province, Nanjing, China

**Keywords:** Glioblastoma, Sensitivity index, PI3K, MTH1, TH588

## Abstract

**Background:**

Glioblastoma multiforme (GBM) is the most common and lethal type of primary brain tumor. More than half of GBMs contain mutation(s) of PTEN/PI3K/AKT, making inhibitors targeting the PI3K pathway very attractive for clinical investigation. However, so far, PI3K/AKT/mTOR inhibitors have not achieved satisfactory therapeutic effects in clinical trials of GBM. In this study, we aimed to develop a high-throughput screening method for high-throughput identification of potential targeted agents that synergize with PI3K inhibitors in GBM.

**Methods:**

A Sensitivity Index (SI)-based drug combination screening method was established to evaluate the interactions between BKM120, a pan-PI3K inhibitor, and compounds from a library of 606 target-selective inhibitors. Proliferation, colony and 3D spheroid formation assays, western blotting, comet assay, γ-H2AX staining were used to evaluate the anti-glioma effects of the top-ranked candidates. The drug combination effects were analyzed by the Chou-Talalay method.

**Results:**

Six compounds were successfully identified from the drug screen, including three previously reported compounds that cause synergistic antitumor effects with PI3K/mTOR inhibitors. TH588, an putative MTH1 inhibitor exhibited significant synergy with BKM120 in suppressing the proliferation, colony formation and 3D spheroid formation of GBM cells. Further investigation revealed that both DNA damage and apoptosis were markedly enhanced upon combination treatment with TH588 and BKM120. Finally, activation of PI3K or overexpression of AKT compromised the anti-glioma efficacy of TH588.

**Conclusions:**

The screening method developed in this study demonstrated its usefulness in the rapid identification of synergistic drug combinations of PI3K inhibitors and targeted agents.

## Background

Glioblastoma multiforme (GBM) is the most common and lethal type of brain cancer in adults. Current first-line therapy for GBM is a combination of surgery, chemotherapy and radiotherapy [1]. Despite such aggressive treatment, the overall survival of treated GBM patients is still shorter than 15 months [2], so the development of novel and effective therapy for GBM is a dire need. However, in the last decade, most clinical trials evaluating new therapeutics in GBM have encountered failures. Major issues need to be solved in the development of future anti-GBM therapy including but not limited to: the insufficient delivery of drug to the brain, genetically heterogeneous nature of GBM, and aberrant activation of multiple signal pathways [3].

The phosphatidylinositol 3-kinase (PI3K) pathway affects several cellular processes that are essential for cancer progression, including cell growth, survival, motility, DNA repair and metabolism [4]. In GBM, several components of the PI3K pathway are frequently mutated. According to the TCGA GBM project, the frequency of genetic alterations in the PI3K pathway, such as mutation or amplification of EGFR, activated mutations of PI3CA (p110) or PIK3R1 (P85), and loss of PTEN, were estimated to occur in up to 88% of GBMs [5]. Therefore, targeting PI3K and its downstream effectors, such as AKT and mTOR, seems to be a promising strategy for treating GBM. Indeed, a variety of targeted agents in PI3K pathways are currently under evaluation in different stages of clinical trials [6].

Unfortunately, most agents targeting the PI3K-AKT-mTOR pathway have failed to achieve satisfactory effects in the treatment of GBM so far [7, 8]. Modest monotherapy activity, unexpected statistically significant toxicity, and limited benefits in survival have been observed for PI3K inhibitors [9–11]. Thus, deep insights into the bottleneck of the anti-glioma effect of PI3K inhibitor treatment are needed. For example, recent research showed that drug resistance is likely driven by compensatory activation of alternative pathways and reorganization of protein networks [12]. Moreover, given the importance of the PI3K pathway in GBM genesis and development as well as the high incidence of mutations in this pathway, novel strategies for more effective combination regimens should be explored to enhance the anti-GBM effect of PI3K inhibitors. Importantly, synergistic combination therapies can improve efficacy, reduce the dose of individual drugs, and avoid potential drug toxicity [13].

In this study, we aimed to develop a method for efficiently identifying targeted agents that work with PI3K inhibitors in synergy to suppress the growth of GBM cells in a high-throughput format. We selected BKM120, a pan-PI3K inhibitor of all catalytic subunit isoforms of class I PI3Ks (p110α, p110β, and p110γ). Interestingly, BKM120 can cross the blood–brain barrier relatively easy because its brain penetration is not restricted by ABCB1 and ABCG2, the two drug-efflux transporters responsible for elimination of most exogenous compounds in the brain. Moreover, invasion to the surrounding tissue is an important feature of GBM and that could be effectively inhibited by BKM120. Therefore, BKM120 represents an attractive candidate drug for GBM treatment [14–17].

To find the agents may sensitize the anti-glioma effect of BKM120 with known targets, we chose a drug library composing of target selective small molecular inhibitors. These small molecules targeting signaling proteins involved in several well-described signaling pathways, including neuronal signaling, metabolism, epigenetics, DNA damage, etc.

Lastly, it would be a laborious work if we want to identify drug combinations with synergistic effects from hundreds of compounds using the conventional dose–response matrix method. Instead, we developed a novel strategy that only the effect of one a fixed concentration of BKM120 need to be tested. This strategy allows high-throughput screening and quick discovery of potential useful combination therapies for GBM treatment.

## Methods

### Cell culture

Human cell lines SNB19, LN229, LN18, U118MG, T98G, A172, HEK293T, Hela and H460 were obtained from the Cell Bank of the Chinese Academy of Sciences (Shanghai, China). U87 and U251 cells were a gift from Dr. X. Hou (China Pharmaceutical University, Nanjing). HEK293T and H460 was maintained in RPMI 1640 (Gibco-Invitrogen, CA, USA) supplemented with 10% fetal bovine serum (FBS, VACCA, Shanghai, China) and penicillin (100 U/mL)-streptomycin (100 µg/mL). All other cell lines were maintained in DMEM (Gibco-Invitrogen) with 10% FBS and penicillin (100 U/mL)-streptomycin (100 µg/mL). All the above cell lines were authenticated by Biowing Biotech (Shanghai, China).

### Antiproliferation assays

To assess the antiproliferative activity of the targeted agents, cells were seeded in 96-well plates at a density of 1200 (U251) or 2000 (all other cell lines) cells per well and were allowed for growth for 24 h. Subsequently, cells were treated with different concentrations of BKM120, either alone or in combination with different concentrations of another targeted agent. Cellular metabolic activity was assessed after 96 h treatment by incubation with Alamar Blue solution (Yeasen, Shanghai, China) for another 4 h, following measurement of fluorescence at 534/584 nm using an Fluoroskan Ascent FL analyzer (Thermo Fisher, CA, USA).

### Colony formation and 3D spheroids for-mation assay

To assess the impact of drug treatment on clonogenicity of GBM cells, 400 cells per well were seed-ed in 12-well plates for 24  h prior to treatment of BKM120, targeted agent, or a combination of both. The drug(s) were added after cell-seeding in the second day, and cells were exposed in the drug-containing medium hereafter with medium refreshing each 3 days. Twelve days after incubation, the colonies were washed with phosphate-buffered saline (PBS) and fixed with 4% paraformaldehyde for 15 min and stained with a Giemsa Stain solution (Yeasen) for 30 min. For the 3D spheroids formation assay, 400 cells per well were seeded in an ultra-low binding 96-well plates with a round bottom (Costar, MA, USA). After 9 days of treatment, digital images of the spheroids were photographed using a phase contrast microscope. The volumes of 3D spheroids were calculated by formula (V = 0.5 * Length * Width^2^) based on the major and minor axial length (Length: major axial length; Width: minor axial length).

### Drug combination screening

A target-selective inhibitors library consisted of 606 target selective small molecule inhibitors (#L3500) were purchased from Selleck Chemicals. U251 cells were seeded in 96-well plates at a density of at 2000 cells per well 24  h prior to screening. The drug screen was performed in following steps: First, cells were treated with DMSO (0.2%), BKM120 (1 μM), agent from the drug library (10 μM), and combination of BKM120 and targeted agent. SI values were calculated as the formula given in Fig. 2a. As a result, 110 targeted agents exhibited excellent synergies with BKM120 (SI ≥ 0.12) and were selected for the further validation. Next, the drug sensitivity for U251 of 110 candidate drugs was reassessed as same as the first step and SI was measured again. Finally, six top ranked compounds in the second step were chosen for further assessment of their antiproliferative effects at concentrations of 10 μM or 2 μM in the presence of 1 μM BKM120 across 8 GBM cell lines, and those continuously achieved high SI values were chosen for further investigation.

### Western blotting

Cells were lysed and homogenized in RIPA buffer, and the protein concentrations were determined using a BCA Protein Assay Kit (Thermo Fisher, CA, USA). Protein samples were separated using 10% sodium dodecyl sulfate–polyacrylamide gels, transferred to PVDF membranes (Merck Millipore, MA, USA) and the membrane was blocked with 3% bovine serum albumin for 1 h. The blots were incubated with specific primary antibodies for 2 h at room temperature or 4 °C overnight, and then incubated with corresponding horseradish peroxidase-conjugated secondary antibodies for 1 h. Finally, it was visualized using an ECL kit (Beyotime, Shanghai, China) and the density of the immunoreactive bands was analyzed using Imager software (Tanon, Shanghai, China). The following primary antibodies used were: pAKT (Ser473) (#4060), AKT (#2920), pS6 (Ser235/236) (#4858), Cleaved-caspase3 (#9661) (all from CellSignaling, Danvers, USA), p4EBP1 (Ser65) (SC293124) (Santa Cruz Biotechnology, CA), MTH1 (abcam, Cambridge, UK), GAPDH (#AC002) (ABclonal, Hubei, China).

### Comet assay

Cells were treated with different drugs for 24 h or 100 mM H_2_O_2_ for 8 min as positive control of DNA damage. Then all cells were harvested, washed and resuspended in PBS at a concentration of 2 × 10^6^ cells/ml. Next, normal melting agarose was dissolved in the above cell suspension to generate a 0.75% agarose mixture. 100 μl and 450 μl cell-agarose mixture were separately added to a fully frosted slide with a 10 min interval to form two layers in the slide and were kept at 4 °C for 30 min. After removing the coverslip, the slide was incubated in lysis buffer (10 mM Tris pH 10.0, 2.5 M NaCl, 0.1 M EDTA2Na, 10% DMSO and 1% Triton X-100) at 4 °C overnight. Then the slide was subjected to electrophoresis in Mini Horizontal Cells (BioRad, CA, USA) filled with ice-cold alkaline electrophoresis buffer (0.3 N NaOH, 1 mM EDTA) at 300 mA and 25 V for 30 min. Soaking in neutralization buffer (0.4 M Tris–HCl, pH 7.5) for 5 min, the slide was counter-stained with 5 mg/ml DAPI (Yeasen). Images were acquired by a Zeiss LSM 510 confocal microscope and were analyzed using a Comet Score software (CASP, CASPLab).

### γ-H2AX staining

Cells received treatment for 3 days were harvested and fixed in 75% ice cold ethanol and kept at − 20 °C overnight. Cells were spun down and washed with ice-cold PBS containing 2% FBS for three times. The cell suspension was incubated with an Anti-phospho-histone H2AX (Ser139) (BioLegend Cat. No. 613404, CA, USA) antibody for 1 h at room temperature. After a brief wash, 10,000 stained cells were collected and analyzed by a BD FACS Verse flow cytometer.

### Laser confocal microscope observation

U251 cells were incubated with TH588 for 48 h on the glass slide, then washed with PBS for three times, and fixed in 4% paraformaldehyde about 20  min, washed three times with PBS containing 0.1% Triton X-100. Then the cells were stained with α-tubulin-tracker red (Beyotime Cat. No. C1050) for 60 min in darkness. A laser scanning confocal microscope (Zeiss LSM880 with NLO & Airyscan) was used to observe the alterations of cytoskeleton proteins. Excitation and emission wavelength were 488  nm and 530  nm, respectively.

### shRNA construction and MTH1 knockdown

MTH1 shRNA sequences were designed by an online tool from Sigma-Aldrich website or obtain from literature. Two MTH1 targeting sequences: shRNA#1 5′-GAAATTCCACGGGTACTTC-3′ and shRNA#2 5′-CGACGACAGCTACTGGTTT-3′ were synthesized and subcloned into a pLKO.1-TRC vector via Age1/EcoRI restriction sites. For transfection, the above plasmids, empty vector together with the packaging plasmids pVSVg (#8454, Addgene) and psPAX2 (#12260, Addgene) were transfected into HEK293T cells at approximately 60% confluence using polyethylenimine (PEI) for 48 h. The culture medium of U251 cells was replaced with the above lentivirus-containing supernatants with the addition of 1:1000 of polybrene and was centrifuged for 2 h at 37 °C for viral infection. After puromycin selection for 3–7 days, the infected cells were collected for validation of MTH1 knockdown.

### Cell apoptosis assay by flow cytometry

Cells received drug treatment for 24 h were collected, washed with ice-cold PBS, and evaluated for apoptosis by double-staining with FITC and PI with an Apoptosis Detection Kit (Yeasen Biotech). Stained cells were analyzed with a FACS Calibur flow cytometer and the obtained data were analyzed by FlowJo 7.6.

### Analysis of PTEN expression-drug sensitivity interactions from public database

We took advantage of the Wooster cell line dataset which consists of over 300 cell lines from 19 cancer types (also see https://cabig.nci.nih.gov/caArray_GSKdata/) for analysis of the correlation between PTEN expression and their sensitivities to PI3K inhibitors. All cell lines were divided into three groups: resistant, intermediate sensitive and sensitive according to their IC50 upon treatment of BKM120 or GSK1059615. The PTEN expressions of all cell lines were obtained from Oncomine (https://www.oncomine.org/).

### Statistics

All experiments except the drug screen and the Western Blotting were performed at least three times independently. Western blotting experiments were performed at least twice independently. The data are expressed as the mean ± SD. Statistical analyses were performed with GraphPad Prism 5.0 software (San Diego, CA, USA). The isobologram analysis of the two drug combinations was performed using CompuSyn 2.0 software (ComboSyn, Inc., Paramus, NJ, USA) [18]. Statistical significance was determined using the two-tailed Student’s *t* test unless otherwise mentioned, with the following *P* values considered significant: *P < 0.05; **P < 0.01; ***P < 0.001.

## Results

### BKM120 blocked PI3K-AKT signaling and exhibited cell line-dependent anti-glioma effects

**Fig. 1:**
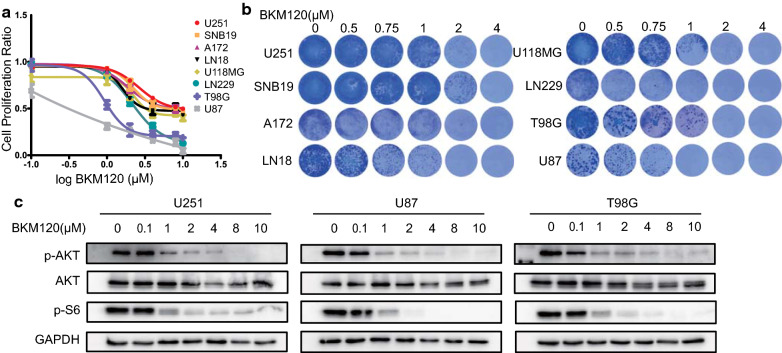
Evaluation of the anti-glioma effect of single agent BKM120. **a** The antiproliferative effect of BKM120 as single agent treatment in eight GBM cell lines. Cell viability was measured with Alamar Blue. Data are presented as percentages relative to the vehicle control. **b** Images of colonies formed by eight GBM cell lines incubated with different concentrations of BKM120 for 14 days followed by Giemsa stain solution on the last day of incubation. **c** Western blot analysis showing blockage of PI3K pathway signaling by BKM120 in three cell lines. Three GBM cell lines were incubated with different concentrations of BKM120 for 24 h

We first investigated the antiproliferative effect of BKM120 using cell viability and colony formation assays across eight GBM cell lines. BKM120 exhibited general growth inhibitory effects in a dose-dependent manner, but limited responsiveness was observed for several cell lines, such as U251, compared with sensitive cell lines like U87 or T98G (Fig. 1a, b). Next, we selected BKM120 sensitive and insensitive cell lines for further investigation of signaling pathway perturbation. Exposure of U251, U87 and T98G cells to BKM120 resulted in suppression of AKT and S6 phosphorylation in a dose-dependent manner, suggesting that the PI3K-AKT signaling was sufficiently blocked even in the BKM120 insensitive cell line (Fig. 1c).

PTEN deletion or mutation is a critical event frequently occurred during GBM development. More than half of the GBM cell lines harbored mutations in the PTEN gene (Additional file 1: Figure S1A), and this could affect the activities of PI3K inhibitor as an anti-cancer agent [19]. We took advantage of the PTEN WT and KO isogenic LN18 cell lines that our lab established previously to investigate the relationship between the PTEN status and the anti-glioma effect of BKM120. As shown in Additional file 1: Figure S1B, PTEN loss did not confer sensitivity to BKM120 in LN18 cells. Furthermore, we analyzed the Wooster cell line dataset which comprise over 300 cell lines treated with 19 drugs. Again, whether these cell lines were sensitive or resistant to BEZ235 or GSK1059615, another pan PI3K inhibitor, were independent of the levels of PTEN expression (Additional file 1: Figure S1C). We also calculated Spearman’s correlation between PTEN protein expression (data obtain from the cell line reverse-phase protein array in the Cancer Cell Line Encyclopedia project) and IC50 (obtained from the Genomics of Drug Sensitivity in Cancer project). We found that low PTEN expression even confers marked increased resistance instead of sensitivity to BKM120 treatment, for a negative (P < 0.05) correlation between the expression of PTEN and the IC50 of another PI3K inhibitor AS605240 was observed (Additional file 1: Figure S1D).

### Drug combination screening identified compounds synergizing anti-glioma effect with BKM120

**Fig. 2:**
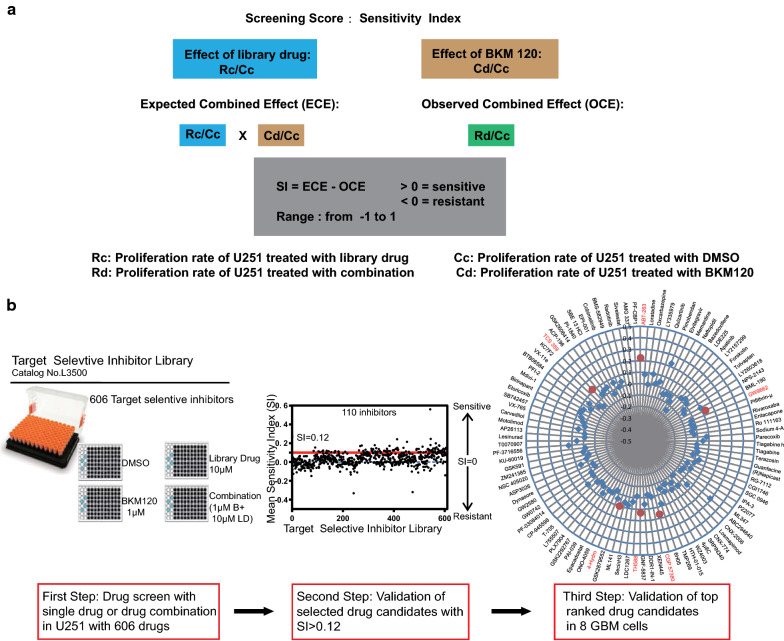
Calculation of Sensitivity Index (SI) and the procedure of drug screening. **a** Formula to calculate the SI value for identification of potential targeted agents that synergizing the anti-glioma effect of BKM120. SI scores > 0 indicate synergy. **b** Workflow of target selective inhibitor screen assays, confirmations, and retesting of compounds. Drug pairs were screened in combination over fixed concentrations. Cell proliferation over 72 h in the presence of drug, relative to vehicle, was determined with Alamar Blue. Scatter plot indicating the number of inhibitors where strong synergy was observed. The cutoff for strong synergy was SI ≥ 0.12. Second round screen results displaying 110 (SI ≥ 0.12) drug measurements. Radial drug interaction plot displaying the 110 pairwise drug-BKM120 measurements. The distance from the center indicates the Sensitivity Index. SI for selected hits are indicated

Evaluation of the drug combination effect can be carried out via various methods and models. However, most of them are based on a dose response matrix, e.g., the Highest Single Agent (HSA) and Bliss independence models [20–22], but such approaches are laborious when screening hundreds of drugs. To simplify this process, we established a method for high-throughput drug combination screening in which a Sensitivity Index (SI) score was introduced to quantify the influence of the addition of another drug. SI was defined as the difference between observed combined effect and expected combined effect which is calculated as the product of the relative proliferation ratio of cells treated with BKM120 and a certain library drug (Fig. 2a). The expected combined effect is estimated as a minimal additive effect of two drug because it is based on an assumption that cells only received BKM120 and a library drug sequentially and no residual effect of BKM120 could affect the following treatment. This formula was also used for calculation of the influence of siRNA-induced knockdown of gene expression on drug sensitivity [23]. The SI scores ranged from − 1 to + 1, with positive values indicating BKM120-sensitizing effects.

From the above results, we determined that 1 μM BKM120 was an appropriate concentration for the combination drug screen, and the U251 cell line was chosen for its insensitivity to BKM120. We took advantage of a drug library containing 606 highly selective small molecule inhibitors targeting multiple cancer-related signaling pathways (Additional file 2: Figure S2). To calculate the SI for individual drugs in the drug library, U251 cells received the following treatments: vehicle (DMSO), 1 μM BKM120, 10 μM library drug and a combination of 1 μM BKM120 and 10 μM library drug (Fig. 2b).

The first step resulted in the identification of 110/606 (1.8%) inhibitors with SI ≥ 0.12, a threshold at which potential BKM120-sensitizing effects were expected. In the second step, the antiproliferative activity of these inhibitors was then re-assessed at 10 μM on U251 ± 1 μM BKM120 to confirm the candidates identified in the first step. Finally, the 6 top ranked compounds displaying relatively strong synergistic effects with BKM120 in the second step were chosen for further assessment of their antiproliferative effects at 10 μM and 2 μM in the presence of 1 μM BKM120 across 8 GBM cell lines (Fig. 2b).

### Validation of the synergism between BKM120 and the candidate agents

**Fig. 3:**
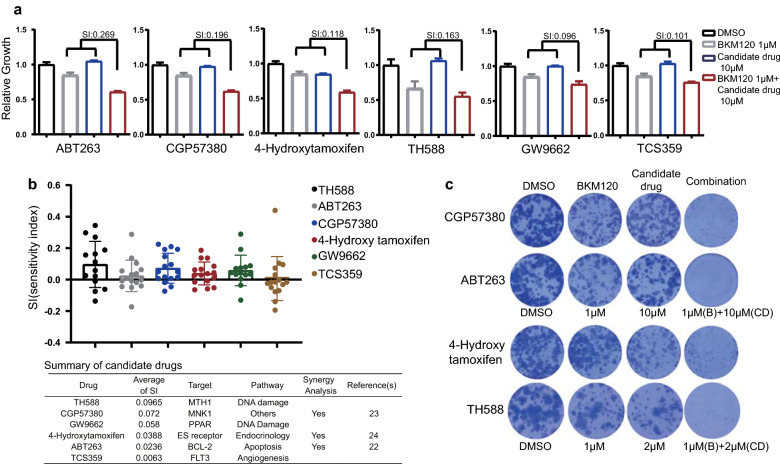
Validation of the BKM120 synergizing effects of candidate agents. **a** Cell viability of U251 cells upon treatment with BKM120 in the presence and absence of a fixed concentration of 6 candidate compounds. **b** Distribution of SI values of multiple GBM cell lines received treatment of BKM120 and targeted agents at different concentrations (2 μM, 10 μM) and a summary of top ranked 6 targeted agents **c** Images of colonies formed by U251 cells incubated with library inhibitors ± BKM120 for 14 days and Giemsa stained on the last day of incubation

For the six top candidates selected above, SI were re-assessed to ensure their abilities to sensitize cells to the antiproliferative effect of BKM120. All candidate compounds had a BKM120-sensitizing effect (SI > 0), and five of them exhibited a strong sensitizing effect (SI > 1.0) (Fig. 3a). Next, we assessed the antiproliferative effect of the combination treatment of eight GBM cell lines. Although differentiated responses were observed across different cell lines and across different treated concentrations, the mean SI of all compounds were all above 0, suggesting an overall BKM120-sensitizing effect of these candidates. A brief summary of the top six candidates were given (Fig. 3b). Finally, the top four candidate compounds, TH588 (an MTH1 inhibitor), CGP57380 (an MNK1 inhibitor), GW9662 (a PPAR inhibitor) and 4-hydroxytamoxifen (an estrogen receptor inhibitor), were chosen for further evaluation. As shown in colony formation assays, these compounds almost eliminated all colonies of U251 in the presence of BKM120, while a single drug or BKM120 failed to show such a strong effect (Fig. 3c). Among them, ABT263, CGP57380 and 4-hydroxytamoxifen have already been reported for their synergistic effects in combination with BKM120 [24–26]. Altogether, this evidence supports that our proof-of-principle screening method is useful to identify potential drug combinations that have synergistic interactions with BKM120.

### Characterization of the anti-glioma effect of the combination treatment of BKM120 and TH588

**Fig. 4:**
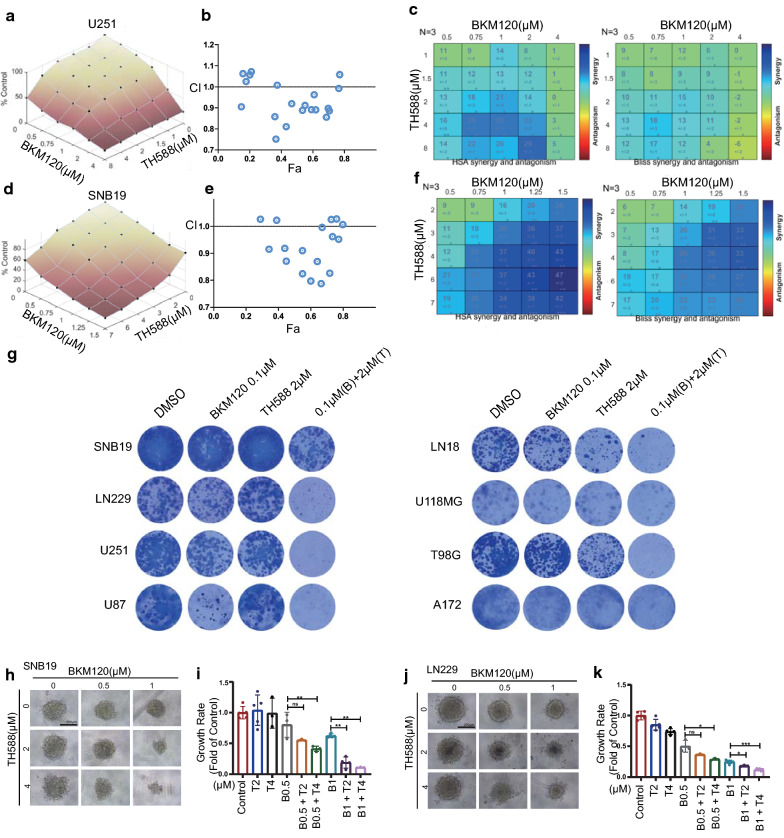
Characterization of the anti-glioma activity of a combination treatment of BKM120 and TH588. **a** Single and combinatorial titration of BKM120 and TH588 in a 3-day growth assay in U251 cell lines. **b** Analysis of the BKM120 and TH588 interaction and the resulting Fa-CI plots (fraction affected Combination Index). For U251, most Combination Index values fell below the CI = 1 line, which indicates synergy of the combination of BKM120 and TH588. **c** Synergy plots generated by Combenefit showing the interaction between BKM120 and TH588. Analysis of interaction resulted in HSA (high single agent) values and Bliss values (n = 3, technical replicates), indicating synergistic efficacy as calculated from expected and observed growth inhibition. HSA and Bliss values > 0 indicate synergistic effects. **d**–**f** Same as (**a**–**c**) but for LN229 cells. **g** Images of colonies formed by several GBM cell lines incubated with TH588, BKM120, or a combination of both for 14 days followed by Giemsa Stain solution on the last day of incubation. **h–k** Three-dimensional (3D) imaging of DMSO or matrix doses of drugs in neurobasal medium on day 15 at 10× magnification and 250 ms exposure. The scale bar is 250 µm

Because TH588 was the top candidate and has not yet been reported elsewhere for its combined effect with BKM120, it was chosen for further investigation. U251 and SNB19 cells were treated with a 5-by-5 dose titration matrix of TH588 and BKM120, and the viability of the cells was determined. As shown in Fig. 4a, d, the expected versus observed dose–response surface plot of the 5-by-5 matrix viability data demonstrated synergistic effect of two agents against U251 and SNB19 GBM cells. In addition, the combination treatment led to marked reduced viability of GBM cells. Moreover, most dots (each dot represents a combination of a specific concentration of two agents) had a CI value below 1.0 in the Fa-CI plots, suggesting the interaction between TH588 and BKM120 were synergistic (Fig. 4b, e). We also applied classical synergy models including HSA and Bliss [20–22] to determine the HSA and Bliss values, which are a readout for synergistic inhibition and depict the difference between expected inhibition and observed inhibition. Most of the HSA and Bliss values were above 0, indicating synergistic interactions between the two drugs (Fig. 4c, f). Furthermore, the combination of TH588 and BKM120 also severely suppressed colony formation in eight GBM cell lines (Fig. 4g) and 3D spheroid formation in SNB19 and LN229 cells (Fig. 4h–k), confirming that the effect of the combination of the two drugs exceeds these of each single agent in suppressing the growth and proliferation of GBM cells in different models.

### The combination of BKM120 and TH588 synergistically induces oxidative DNA damage and apoptosis

**Fig. 5:**
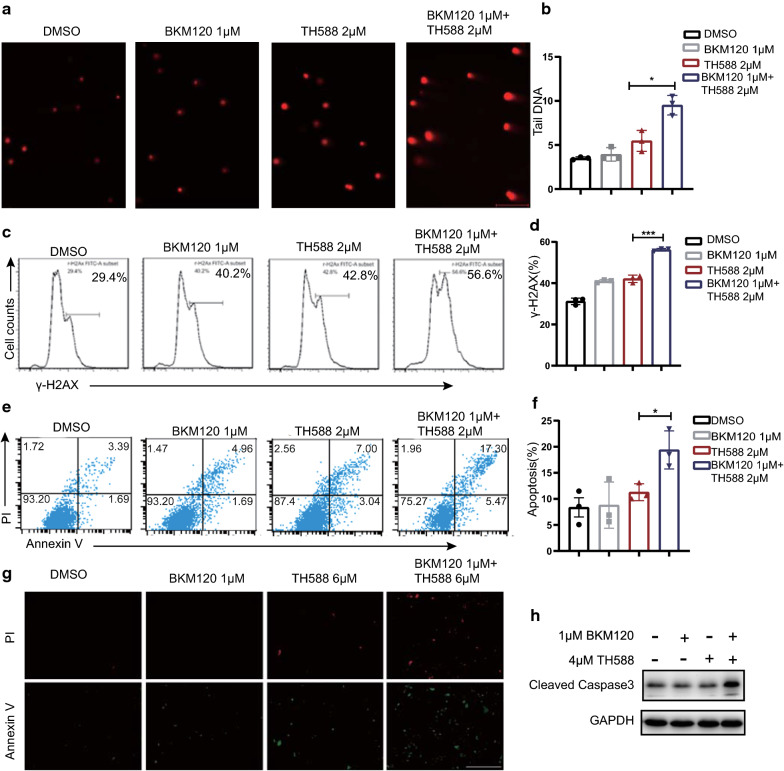
The combination of BKM120 and TH588 synergistically induces oxidative DNA damage and apoptosis. **a** Comet assay of U251 cells following treatment with BKM120, TH588 or the combination for 24 h. H2O2 was used as a control. **b** Quantification of comet tail moment. Tail moment, calculated as  % DNA in the tail multiplied by the tail length. Values represent the average ± SD from three independent experiments (> 25 comets per experiment, **P < 0.01). **c** Flow cytometry assay of H2AX phosphorylation in U251 cells following treatment with increasing concentrations of TH588 for 24 h. **d** Quantification of γ-H2AX-positive cells from B (*P < 0.05, **P < 0.01). **e** Flow cytometric analysis of  % apoptotic U251 cells (stained by annexin V/PI) following BKM120, TH588 or combination pretreatment for 24 h. **f** Quantification of apoptotic cells from **f**; shown is the average ± SD, n = 3 (*P < 0.05, **P < 0.01). **g** U251 cells treated with BKM120, TH588 or a combination of both for 48 h, and then subjected with co-staining with annexin V and PI. Cells were observed under a fluorescent microscope and representative photos were given. Scale bars = 0.2 mm. **h** U251 cells received treatment of BKM120,TH588 or a combination of both for 48 h were immunoblotted with antibodies of cleaved caspase-3 and GAPDH

TH588 has been reported to cause DNA damage by increasing 8-oxodG or 2-OH-dA incorporation into DNA, leading to DNA base mispairing, mutation and cell death [27]. Interestingly, previous works suggest that inhibition of PI3K interferes with DNA synthesis or repair through nucleoside depletion [28] so there might be a link between BKM120 and TH588. To reveal the synergistic mechanism, we used a comet assay, a sensitive method for detecting 8-oxodG or 2-OH-dA at the single-cell level to evaluate the DNA damage caused by the combination treatment [29]. The significant elongated tailing ratio of combination treatment relative to control or single agent treatment suggests that more abundant DNA-damaged fragments and accumulation of 8-oxodG in DNA were presented after treatment of both MTH1 and PI3K inhibitor (Fig. 5a, b).

The nucleosomal histone protein H2AX is rapidly phosphorylated at serine 139 (γ-H2AX) at the double strand break (DSB) site [30], so γ-H2AX foci formation is usually used to quantify DSBs. Therefore, we reasoned that the PI3K inhibitor also enhanced the DSB damage of DNA caused by TH588. To confirm that, GBM cells treated with BKM120 and/or TH588 was stained with γ-H2AX and analyzed by flow cytometry. As shown in Fig. 5c, d and Additional file 3: Figure S3, the γ-H2AX-positive fraction of U251 cells increased significantly upon treatment with BKM120 and TH588 compared with that in cells that received single-agent treatment, suggesting that the combination of PI3K and MTH1 inhibitors dramatically elevates DSB levels in GBM or lung cancer cells. Accumulating evidence suggests that PI3K pathway is also involved in DNA replication and genome stability [28]. Therefore, PI3K might be an attractive target for combining with targeted agent related to DNA repairing pathway [31].

Moreover, comparing with the independent treatment of BKM120 and TH588, the combination treatment significantly increased the Annexin V-positive apoptotic fraction of cells (Fig. 5e–g and Additional file 5: Figure S5), and also caused marked elevation of cleaved caspase3.

On top of the MTH1 inhibition, alternative mechanisms of TH588 have been reported as its anti-cancer effects including tubulin depolymerization and AKT signaling downregulation [32, 33]. To confirm it, we investigated the spindle morphology of mitotic cells upon treatment of 5 and 10 µM of TH588. Indeed, TH588 caused disruption of spindle formation and interrupted the separations of duplicated centrosomes (Additional file 3: Figure S3). In addition, treatment of TH588 also caused reduction of phosphor Akt (S473) and its downstream component 4EBP1 (S65) in U251 and U87 cells (Additional file 3: Figure S3). Together these evidence provide additional information for TH588 mediated GBM cell death besides MTH1 inhibition.

### The PI3K/AKT signaling is a determinant of the responsiveness to MTH1 inhibition

**Fig. 6:**
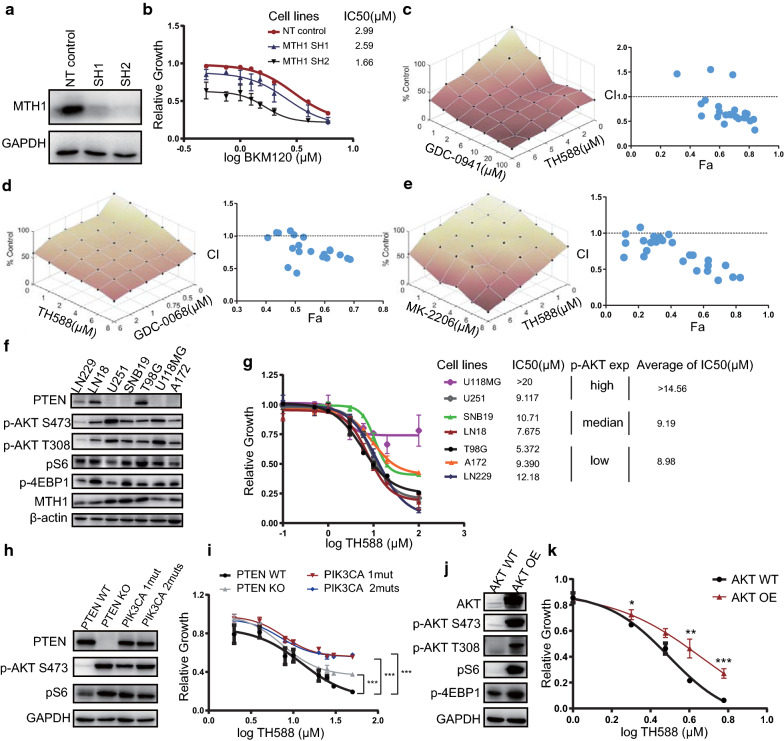
The PI3K/AKT pathway is a determinant of the responsiveness of GBM cells to MTH1 inhibition. **a** Lysates were collected at multiple time points from U251 cells expressing either a control shRNA plasmid or an MTH1 shRNA to confirm MTH1 knockdown. **b** Decreased expression of MTH1 increased the sensitivity of U251 cells to BKM120. **c** (Left) Single and combinatorial titration of GDC-0941 and TH588 cells in the 3 days growth assay in U251 cell lines. GDC-0941 is a highly specific pan-PI3K inhibitor. (Right) Analysis of GDC-0941 and TH588 interaction and the resulting Fa-CI plots. **d** (Left) Single and combinatorial titration of GDC-0068 and TH588 cells in the 3 days growth assay in U251 cell lines. GDC-0068 is a novel, highly selective, orally available ATP-competitive pan-AKT inhibitor. (Right) Analysis of GDC-0068 and TH588 interaction and the resulting Fa-CI plots. **e** Same as (**d**), but for MK2206, a potent, oral allosteric AKT inhibitor. **f** Basal levels of PTEN, p-AKT473, p-AKT308, p-S6 and p-4EBP1 in a panel of eight GBM cells were determined by Western blot analysis. **g** A panel of eight GBM cells was incubated with different concentrations of BKM120 for 72 h. **h** Levels of PTEN, p-AKT473, p-AKT308, p-S6 and p-4EBP1 in LN229, LN229 with inducible PTEN knockdown, LN229 with PIK3CA mutant 1 and 2 were determined by Western blot analysis. **i** Cell viability assay in LN229, LN229 with inducible PTEN knockdown, LN229 with PIK3CA 1 mut (p.E542K) or LN229 with PIK3CA 2 muts (p.N345K and p.E542K) treated with TH588. Error bars represent the standard error of the mean (SD). **j** Western blot analysis of AKT overexpression in HeLa cells. **k** Cell viability assay in HeLa cells with AKT overexpression treated with TH588. Error bars represent the standard error of the mean (SD)

To assure that the antiproliferative activity of TH588 is due to its selective targeting of MTH1 but not an off-target effect, stable MTH1 knockdown U251 together with control cells transduced with only empty vector control were established. The expression of MTH1 in two MTH1 knockdown cell lines was validated by Western blotting (Fig. 6a). As shown in Fig. 6b, MTH1-silenced U251 cells were generally sensitive to BKM120, and the IC50 of two MTH1-silenced cells (2.59 and 1.63) were lower than that of control cells (2.99).

To further investigate whether the TH588 synergizing effect with PI3K inhibitor only limit to BKM120, we also assessed the combined anti-glioma effect of TH588 with other PI3K and AKT inhibitors. For GDC-0941, another pan-PI3K inhibitor, it effectively suppressed the proliferation of GBM cells with TH588, and the Combination Index (CI) values were lower than 1 at most concentrations, suggesting the interaction between two agents were synergistic (Fig. 6c). For GDC-0068 and MK-2206, two highly selective AKT inhibitors, most CI values were also below 1 when they were combined with TH588 (Fig. 6d, e). The above evidence suggested that both PI3K and AKT inhibitors enhanced the antiproliferation effect of MTH1 blockade or knockdown, indicating that the PI3K-AKT pathway activation may be an important determinant for the anti-glioma efficacy of MTH1 inhibition.

To find more evidence supporting this finding, we also checked the levels of PI3K-AKT signaling activation in eight GBM cell lines and categorized them into low, medium or high groups according to the expression levels of p-AKT (Fig. 6f). Next, we performed a proliferation assay by treating these GBM cell lines with TH588 and analyzing the corresponding IC50. Although the number of cell lines used was limited, GBM cells with highly activated PI3K-AKT signaling tended to be more resistant to TH588 (IC50 values for the p-AKT low, medium and high groups were > 14.56, 9.19 and 8.98 µM, respectively, Fig. 6g). Based on this observation, we established LN229 isogenic cell lines with PTEN wild type, PTEN knockout, and PIK3CA carrying constitutively activated mutations (p.N345K and p.E542K) [34] and investigated their responses to MTH1 inhibition. It is known that loss-of-function mutations of PTEN and gain-of-function mutations of PIK3CA can both activate the PI3K-AKT pathway. Indeed, the levels of p-AKT and its downstream component p-S6 were elevated in all three mutant cell lines compared with LN229 parental cells (Fig. 6h). In line with the above results, both PTEN knockout and PIK3CA mutant cells were less responsive to TH588 than their parental cell lines (Fig. 6i). Finally, we transfected a plasmid carrying the intact AKT gene or an empty vector into HeLa cells, and it led to elevation of p-AKT, p-S6 and p-4EBP1 expression in AKT-overexpressing cells relative to empty vector-transfected cells (Fig. 6j). Similarly, AKT-overexpressing cells showed significantly elevated TH588 tolerance compared with that in empty vector-transfected control cells (Fig. 6k). Altogether, we found that the anti-glioma effect of TH588 was determined by the activation of the PI3K-AKT signaling pathway, which may provide an explanation for the synergistic interaction between PI3K and MTH1 inhibitors.

## Discussion

In this study, we developed a simple and efficient screening method for high-throughput identification of agents that synergize with a PI3K inhibitor. The primary screening was based on the Sensitivity Index, which was calculated as the difference between the observed combined effect and expected combined effect. As a result, at least six promising BKM120 synergistic combinations were identified and validated. Among them, three top-ranked candidates, namely, MNK1, estrogen receptor and BCL-2 inhibitors, have been reported previously for their antitumor effects in combination with PI3K/mTOR inhibitors, confirming the usefulness of this screening method. Importantly, in this study, we successfully identified a novel combination of BKM120 and the MTH1 inhibitor TH588, which had a potent anti-glioma effect. Further investigation suggested that the synergy between PI3K and MTH1 inhibitors was not only limited to BKM120 and TH588, but also can be extended to AKT or other PI3K inhibitors together with TH588.

Single-agent PI3K inhibitors failed to exhibit a significant therapeutic response in clinical trials of malignant gliomas for multiple reasons. The data presented here showed that BKM120 alone suppressed the PI3K-AKT pathway as well as arrested GBM cell proliferation, but this effect was dose-dependent and cell line-dependent. In addition, GBMs carrying PTEN mutations or deletions were previously thought to be sensitive to PI3K inhibitors. However, we failed to find that these tumors responded better to PI3K inhibitors than PTEN wild-type GBM. Given the importance of the PI3K-AKT signaling pathway and the fact that PTEN was mutated or deleted in 40–60% of GBMs, it is necessary to explore effective combined treatment to boost the anti-glioma effect of PI3K inhibitors. As a result of the drug screening, six compounds that significantly enhanced the anti-glioma effect of BKM120 in the PTEN-deficient GBM cell line were successfully identified, and the MTH1 inhibitor TH588, which had the highest SI score in the screen, was further studied.

The human mutT homologue MTH1 is a human 8-oxo-dGTPase that removes oxidized bases in the nucleotide pool and DNA, thus avoiding ROS-induced DNA misincorporation, mutations and cell death [27]. In contrast, normal cells repair oxidative damage to DNA via alternative pathways. The severe dependence on MTH1-mediated DNA repair in cancer cells makes MTH1 an attractive target in cancer treatment [35, 36]. Recent reports indicated that cancer cells were also heavily dependent on functional MTH1 to maintain stemness and tumorigenesis [37, 38]. Several small molecular inhibitor of MTH1 including TH588, BAY707 and S-crizotinib were developed and their anti-tumor activities looks promising [27, 39, 40]. For TH588, some researchers questioned its target specificity as this agent also possesses microtubule-modulating activity. Indeed, in our study we also found that treatment of TH588 cause mitotic spindles disruption beside its potent anti-GBM effect in the presence of BKM120. Therefore, TH588 may has other target(s) except MTH1. Interestingly, previous work reported the effectiveness of the combination of TH588 and everolimus, an mTORC1 inhibitor, in the treatment of neuroendocrine cancer cells [32]. However, brain delivery across the blood–brain barrier of everolimus is limited [7]. In this study, we reported the synergy between TH588 and BKM120, a pan-PI3K inhibitor penetrating the blood–brain barrier more efficiently than rapalogs [41].

Some evidence from previous works supports our finding that there are functional interactions between PI3K pathway inhibition and ROS-induced DNA damage repair [27, 28] and PARP-mediated base excision repair [42]. Indeed, we found a significant increase in the proportion of segmented tail DNA and phosphorylated H2AX under combined treatment with TH588 and BKM120, suggesting that the DNA damage repair pathway was indeed affected by the PI3K pathway and that blockade of both causes severe consequences for cancer cells.

## Conclusions

In conclusion, with a simple and efficient screening method, we successfully identified several promising and effective combinations of targeted drugs with BKM120 that potently inhibit GBM proliferation and growth. Among them, an MTH1 inhibitor TH588 excelled other top ranked candidate targeted agents and exhibited a strong synergistic anti-glioma effect with BKM120, thus offering a potential combination therapy for further evaluation in GBM models. Overall, the screening method used in this study provides a useful and reliable tool for finding novel and effective combination therapies for refractory tumors such as GBM.

## Supplementary information

**Additional file 1: Figure S1.** PTEN gene mutations in GBM cells and its impact on drug sensitivities of PI3K inhibitors. (A) Mutations of four essential GBM oncogenes or tumor suppressor genes in eight GBM cell lines. (B) Antiproliferation rate of LN18, LN18 sgRNA negative control, LN18 PTEN knockout #1 and 2 cell lines upon treatment of BKM120. (C) PTEN expression in PI3K inhibitor sensitive, intermediate sensitive or resistant cell lines collection. GSK1059615 is a dual inhibitor of pan-class I PI3K and mTOR kinase. (D) Linear regression of PTEN protein levels and BKM120 sensitivity demonstrated a strong correlative trend in terms of R and p values by Spearman’s rank correlation test (p=0.0256). Data of C and D were obtained from public database (Wooster dataset and Oncomine database).

**Additional file 2: Figure S2.** Summary of the targeting pathways of 606 small molecule inhibitors in the drug library (Selleck #L3500).

**Additional file 3: Figure S3.** Treatment of BKM120 and TH588 caused elevation of γ-H2AX-positive cells. Left: Flow cytometry analysis of γ-H2AX stained LN229 GBM cells following treatment of vehicle (DMSO), BKM120, TH588 and combination of both for 24 h. Right: Quantification of γ-H2AX-positive LN229 cells of each type of treatment in triplicates.

**Additional file 4: Figure S4.** Flow cytometric analysis of apoptotic cells upon treatment of TH588 and/or BKM120. Left: H460 cells were treated with vehicle (DMSO), BKM120, TH588 or combination of both for 24 h and analyzed by flow cytometry for quantification of the fraction of apoptotic cells (pre-stained with annexin V/PI). Right: Quantification of apoptotic fraction of H460 cells received each type of treatment in triplicates.

**Additional file 5: Figure S5**. TH588 disrupts mitotic spindles and causes AKT pathway downregulation. (A) Photomicrographs of mitotic cells treated with DMSO or TH588 for 48 hours showing α-tubulin (red), and chromatin (blue, DAPI). Scale bar = 10 μm. (B) Western blot analysis of components from the AKT pathway were analyzed after 48 h treatment of TH588.

## Data Availability

The analysed data sets generated during the study are available from the corresponding author on reasonable request.

## Abbreviations

GBM: Glioblastoma multiforme
SI: Sensitivity index
TCGA: The cancer genome atlas
CCLE: Cancer cell line encyclopedia
GDSC: Genomics of drug sensitivity in cancer
IC50: Half maximal inhibitory concentration
CI: Combination index
HSA: Highest single agent
BLISS: Bi-level intergrated system synthesis
DSB: Double strand breaks
DMSO: Dimethyl sulfoxide
PBS: Phosphatebuffered saline
