# Supplementary material for: A high-throughput drug combination screen identifies an anti-glioma synergism between TH588 and PI3K inhibitors
Source: Cancer Cell Int. 2020 Jul 23;20:337. doi: 10.1186/s12935-020-01427-0 (PMC7376673; doi:10.1186/s12935-020-01427-0)
Supplement: Supplementary file 1 — Additional file 1: Figure S1. PTEN gene mutations in GBM cells and its impact on drug sensitivities of PI3K inhibitors. (A) Mutations of four essential GBM oncogenes or tumor suppressor genes in eight GBM cell lines. (B) Antiproliferation rate of LN18, LN18 sgRNA negative control, LN18 PTEN knockout #1 and 2 cell lines upon treatment of BKM120. (C) PTEN expression in PI3K inhibitor sensitive, intermediate sensitive or resistant cell lines collection. GSK1059615 is a dual inhibitor of pan-class I PI3K and mTOR kinase. (D) Linear regression of PTEN protein levels and BKM120 sensitivity demonstrated a strong correlative trend in terms of R and p values by Spearman’s rank correlation test (p=0.0256). Data of C and D were obtained from public database (Wooster dataset and Oncomine database). [file 12935_2020_1427_MOESM1_ESM.pdf]

Figure S1

A

Gene Name	U251	SNB19	LN18	LN229	A172	U118MG	T98G	U87
EGFR	wt	wt	wt	wt	focal gain,rearranged	wt	wt	wt
PTEN	mutation	homozygous frameshift	wt	wt	homozygous deletion	mutation	homozygous missense	homozygous splice site
PIK3CA	wt	wt	wt	wt	focal gain	wt	wt	wt
P53	inactivating mutation	homozygous missense	homozygous missense	homozygous missense	wt	homozygous missense	homozygous missense	wt

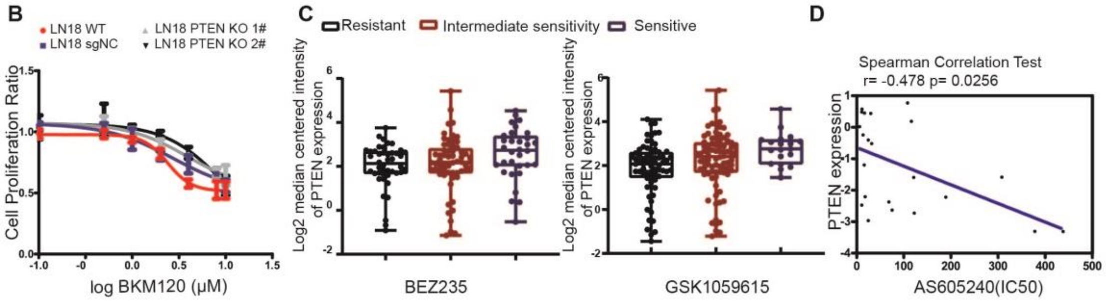

Figure S1. PTEN gene mutations in GBM cells and its impact on drug sensitivities of PI3K inhibitors. (A) Mutations of four essential GBM oncogenes or tumor suppressor genes in eight GBM cell lines. (B) Antiproliferation rate of LN18, LN18 sgRNA negative control, LN18 PTEN knockout #1 and 2 cell lines upon treatment of BKM120. (C) PTEN expression in PI3K inhibitor sensitive, intermediate sensitive or resistant cell lines collection. GSK1059615 is a dual inhibitor of pan-class I PI3K and mTOR kinase. (D) Linear regression of PTEN protein levels and BKM120 sensitivity demonstrated a strong correlative trend in terms of R and p values by Spearman's rank correlation test (p=0.0256). Data of C and D were obtained from public database (Wooster dataset and Oncomine database).
