# Supplementary material for: A high-throughput drug combination screen identifies an anti-glioma synergism between TH588 and PI3K inhibitors
Source: Cancer Cell Int. 2020 Jul 23;20:337. doi: 10.1186/s12935-020-01427-0 (PMC7376673; doi:10.1186/s12935-020-01427-0)
Supplement: Supplementary file 2 — Additional file 2: Figure S2. Summary of the targeting pathways of 606 small molecule inhibitors in the drug library (Selleck #L3500). [file 12935_2020_1427_MOESM2_ESM.pdf]

**Figure S2**

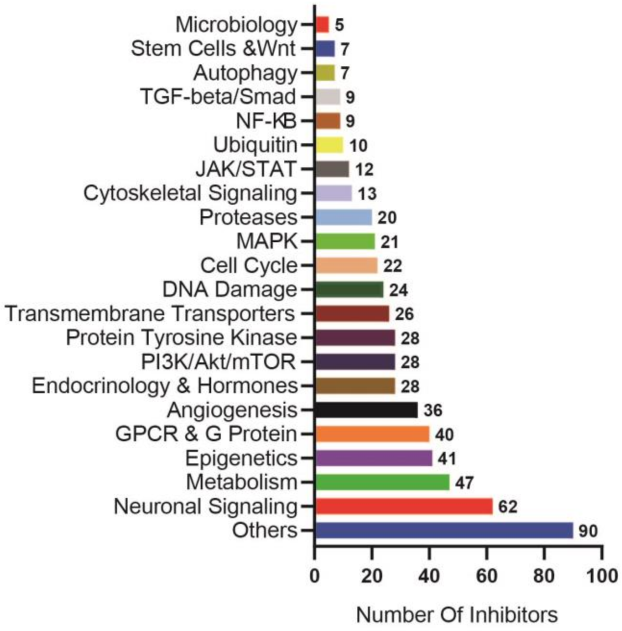


**Figure S2. Summary of the targeting pathways of 606 small molecule inhibitors in the drug library (Selleck #L3500).**
