# Supplementary material for: A high-throughput drug combination screen identifies an anti-glioma synergism between TH588 and PI3K inhibitors
Source: Cancer Cell Int. 2020 Jul 23;20:337. doi: 10.1186/s12935-020-01427-0 (PMC7376673; doi:10.1186/s12935-020-01427-0)
Supplement: Supplementary file 3 — Additional file 3: Figure S3. Treatment of BKM120 and TH588 caused elevation of γ-H2AX-positive cells. Left: Flow cytometry analysis of γ-H2AX stained LN229 GBM cells following treatment of vehicle (DMSO), BKM120, TH588 and combination of both for 24 h. Right: Quantification of γ-H2AX-positive LN229 cells of each type of treatment in triplicates. [file 12935_2020_1427_MOESM3_ESM.pdf]

Figure S3

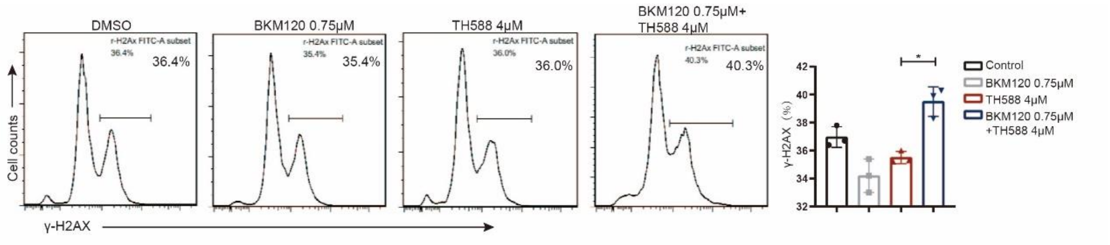

Figure S3. Treatment of BKM120 and TH588 caused elevation of γ-H2AX-positive cells. Left: Flow cytometry analysis of γ-H2AX stained LN229 GBM cells following treatment of vehicle (DMSO), BKM120, TH588 and combination of both for 24 h. Right: Quantification of γ-H2AX-positive LN229 cells of each type of treatment in triplicates.
