# Supplementary material for: A high-throughput drug combination screen identifies an anti-glioma synergism between TH588 and PI3K inhibitors
Source: Cancer Cell Int. 2020 Jul 23;20:337. doi: 10.1186/s12935-020-01427-0 (PMC7376673; doi:10.1186/s12935-020-01427-0)
Supplement: Supplementary file 4 — Additional file 4: Figure S4. Flow cytometric analysis of apoptotic cells upon treatment of TH588 and/or BKM120. Left: H460 cells were treated with vehicle (DMSO), BKM120, TH588 or combination of both for 24 h and analyzed by flow cytometry for quantification of the fraction of apoptotic cells (pre-stained with annexin V/PI). Right: Quantification of apoptotic fraction of H460 cells received each type of treatment in triplicates. [file 12935_2020_1427_MOESM4_ESM.pdf]

Figure S4

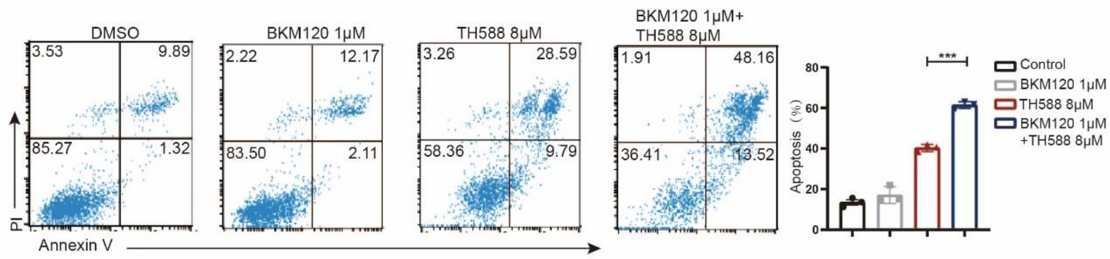

Figure S4. Flow cytometric analysis of apoptotic cells upon treatment of TH588 and/or BKM120. Left: H460 cells were treated with vehicle (DMSO), BKM120, TH588 or combination of both for 24 h and analyzed by flow cytometry for quantification of the fraction of apoptotic cells (pre-stained with annexin V/PI). Right: Quantification of apoptotic fraction of H460 cells received each type of treatment in triplicates.
