# Supplementary material for: A high-throughput drug combination screen identifies an anti-glioma synergism between TH588 and PI3K inhibitors
Source: Cancer Cell Int. 2020 Jul 23;20:337. doi: 10.1186/s12935-020-01427-0 (PMC7376673; doi:10.1186/s12935-020-01427-0)
Supplement: Supplementary file 5 — Additional file 5: Figure S5. TH588 disrupts mitotic spindles and causes AKT pathway downregulation. (A) Photomicrographs of mitotic cells treated with DMSO or TH588 for 48 hours showing α-tubulin (red), and chromatin (blue, DAPI). Scale bar = 10 μm. (B) Western blot analysis of components from the AKT pathway were analyzed after 48 h treatment of TH588. [file 12935_2020_1427_MOESM5_ESM.pdf]

**Figure S5**

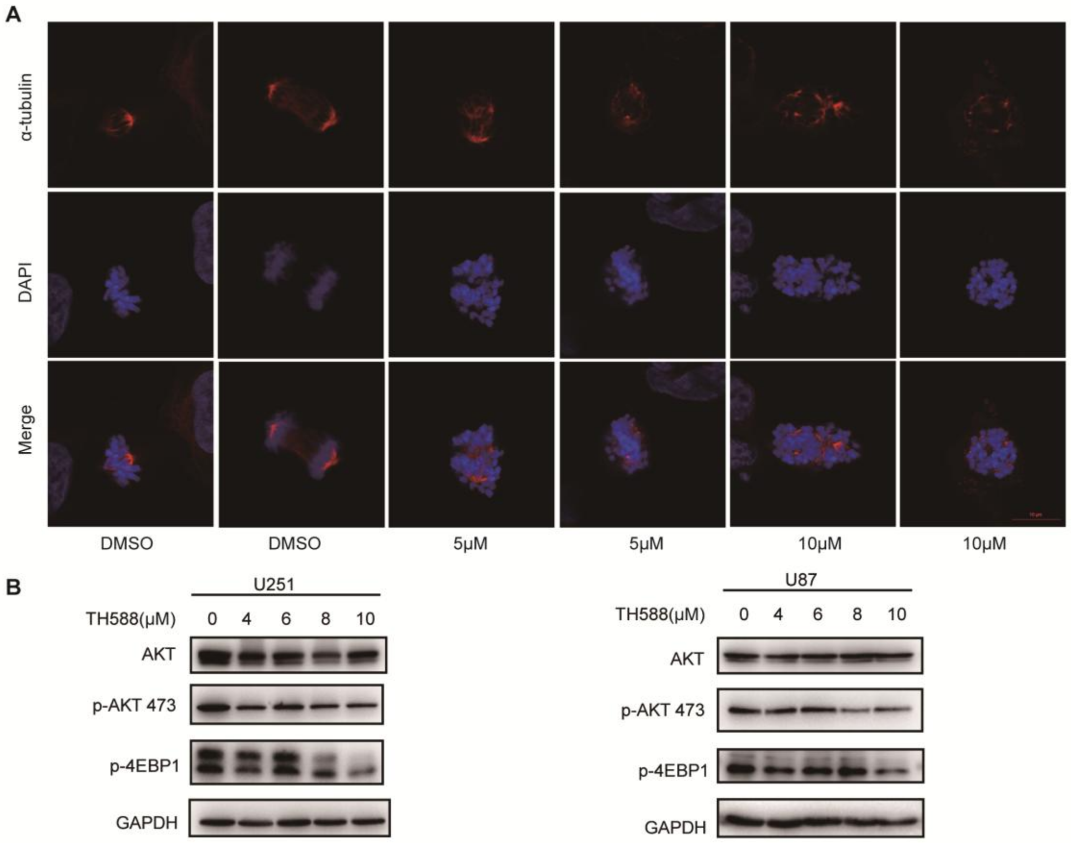


**Figure S5. TH588 disrupts mitotic spindles and causes AKT pathway downregulation.** (A) Photomicrographs of mitotic cells treated with DMSO or TH588 for 48 hours showing α-tubulin (red), and chromatin (blue, DAPI). Scale bar = 10 μm. (B) Western blot analysis of components from the AKT pathway were analyzed after 48 hours treatment of TH588.
